# Maintenance treatment of Uracil and Tegafur (UFT) in responders following first-line fluorouracil-based chemotherapy in metastatic gastric cancer: a randomized phase II study

**DOI:** 10.18632/oncotarget.13922

**Published:** 2016-12-12

**Authors:** Wenhua Li, Xiaoying Zhao, Huijie Wang, Xin Liu, Xinmin Zhao, Mingzhu Huang, Lixin Qiu, Wen Zhang, Zhiyu Chen, Weijian Guo, Jin Li, Xiaodong Zhu

**Affiliations:** ^1^ Department of Medical Oncology, Fudan University Shanghai Cancer Center, Shanghai, China; ^2^ Department of Oncology, Shanghai Medical College, Fudan University, Shanghai, China

**Keywords:** gastric carcinoma, UFT, chemotherapy, maintenance treatment

## Abstract

**Background:**

Maintenance therapy proves to be effective in advanced lung and breast cancer after initial chemotherapy. The purpose of this phase II study was to evaluate the efficacy and safety of Uracil and Tegafur (UFT) maintenance in metastatic gastric cancer patients following the first-line fluorouracil-based chemotherapy.

**Methods:**

Metastatic gastric cancer patients with stable disease or a better response after the completion of first-line chemotherapy were randomized to oral UFT (360mg/m2 × 2 weeks) every 3 weeks until disease progression/intolerable toxicity or to observation (OBS). The primary endpoint was progression-free survival (PFS); the secondary endpoints were overall survival (OS) and safety.

**Results:**

The trial was closed after the interim analysis of the 58 enrolled (120 planned) patients. Median PFS was not improved in the UFT group compared with the OBS group (3.2 months versus 3.6 months, *P* = 0.752), as well as the median OS (14.2 months for both, *P* = 0.983). However, subgroup analysis showed that low baseline hemoglobin (< 120 g/L) was associated with poorer PFS with maintenance therapy (*P* = 0.032), while the normal hemoglobin patients benefit from the UFT treatment (*P* = 0.008). Grade 3 to 4 toxicities in the UFT group were anemia (3.4%), thrombocytopenia (3.4%) and diarrhea (6.9%).

**Conclusions:**

This trial did not show superiority of UFT maintenance in non-selected patients responding to fluorouracil-based first-line chemotherapy. The normal hemoglobin level at baseline is a predictive biomarker for favorable patient subsets from the maintenance treatment.

## INTRODUCTION

Gastric cancer is the third leading cause of cancer-related death worldwide. Nearly 1 million people in the world are diagnosed with gastric cancer every year, and approximately half of them are in China [1]. Fluorouracil-based regimens, such as ECF (epirubicin, cisplatin and 5-fluorouracil), EOF(epirubicin, oxaliplatin and 5-fluorouracil) and XELOX (oxaliplatin plus capecitabine), have been demonstrated to provide survival benefit to patients with advanced or metastatic gastric cancer [2, 3]. However, recurrence or disease progression is still the main cause for treatment failure.

The concept of maintenance therapy was raised up for expectation of further improvement in efficacy and reduction on disease progression after the initial treatment. In non-small-cell lung cancer, continuation maintenance with pemetrexed or gemcitabine was proved to delay progression after the induction therapy with pemetrexed/gemcitabine plus cisplatin [4, 5]. Other anti-cancer drugs with low toxicity could also be considered on maintenance treatment, such as the bevacizumab and tyrosine kinase inhibitors (TKIs) [6, 7]. In breast cancer, maintenance chemotherapy improves PFS but not OS [8]. However, whether the mode of maintenance treatment could be feasible in gastric cancer is unknown.

Uracil and Tegafur (UFT) is an oral 5-fluorouracil (5-FU) compound combining tegafur and uracil in a molar ration of 1:4. Tegafur is metabolized to 5-FU in the liver, and uracil inhibits the main metabolizing enzyme of 5-FU, thereby increasing serum concentration of 5-FU. Oral UFT plus leucovorin (LV) was demonstrated to be equivalent in efficacy with 5-FU/LV in both the adjuvant setting and palliative setting of chemotherapy with the tolerable toxicity [9, 10]. UFT was also suggested as the maintenance therapy after surgical adjuvant chemotherapy in stage III colon cancer [11]. As an oral tablet, UFT has practical advantage in long-term treatment. UFT was recommended as one of the oral agents in treating advanced gastric cancer in Japan [12, 13]. Thus, our randomized phase II trial was conducted to further confirm the efficacy of UFT as the maintenance therapy in patients with metastatic gastric cancer for whom response to fluorouracil-based chemotherapy in the first-line treatment.

## RESULTS

### Patients

The trial was activated on August 2009. On June 2014, after the enrollment of 58 patients, recruitment was stopped as a consequence of the negative outcome of the interim data analysis. This report is consequently on the basis of the data available from each of the 58 eligible patients as of June 2016.

A total of 58 patients were randomly assigned in the study, with 29 assigned to UFT group and 29 assigned to observation group. The demographics and baseline characteristics of the two arms were well balanced (Table 1). Fifty-four patients received EOF regimen in the first-line chemotherapy. The percentages of patients with more than 2 metastatic sites and peritoneal metastases in the observation group were relatively higher than that in the UFT group, respectively 51.7% versus 34.5% and 44.8% versus 24.1%, but there was no statistically significant difference between the two arms (*P* = 0.18 and 0.58).

**Table 1:** Baseline demographic and clinical characteristics of full analysis set

	No. (%)	
Characteristic	UFT group (*n* = 29)	OBS group (*n* = 29)
Age, years		
Median	55	55
Range	23-74	26-70
Sex		
Male	22 (75.9)	19 (65.5)
Female	7 (24.1)	10 (34.5)
ECOG PS		
0	8 (27.6)	8 (27.6)
1	20 (69)	21 (72.4)
2	1 (3.4)	0
Primary lesion		
Gastric	28 (96.6)	29 (100)
Gastroesophageal junction	1 (3.4)	0
Prior gastrectomy	7 (24.1)	8 (27.6)
No. of metastatic sites		
≤ 2	19 (65.5)	14 (48.3)
> 2	10 (34.5)	15 (51.7)
Peritoneal metastases	7 (24.1)	13 (44.8)
Prior chemotherapy regimen		
ECF/EOF	28 (96.6)	28 (96.6)
FOLFOX/XELOX	1 (3.4)	1 (3.4)
Efficacy of prior chemotherapy		
PR	14 (48.3)	14 (48.3)
SD	15 (51.7)	15 (51.7)
Baseline Hemoglobin		
≥ 80 and < 120 g/L	17(58.6)	15 (51.7)
≥ 120 g/L	12 (41.4)	14 (48.3)

Abbreviation: ECOG PS, Eastern Cooperative Oncology Group performance status; PR, partial recession; SD, stable disease.

### Treatment

Patients in the UFT group received 6.3 cycles of medication on average (range 1-44). Eight-three percent of patients were treated over two or more cycles, and 34.5% percent of patients over six or more cycles. There were 6 patients experienced disease progression within one month after the randomization in the UFT group, while there were 4 in the OBS group.

### Efficacy

**Figure 1:**
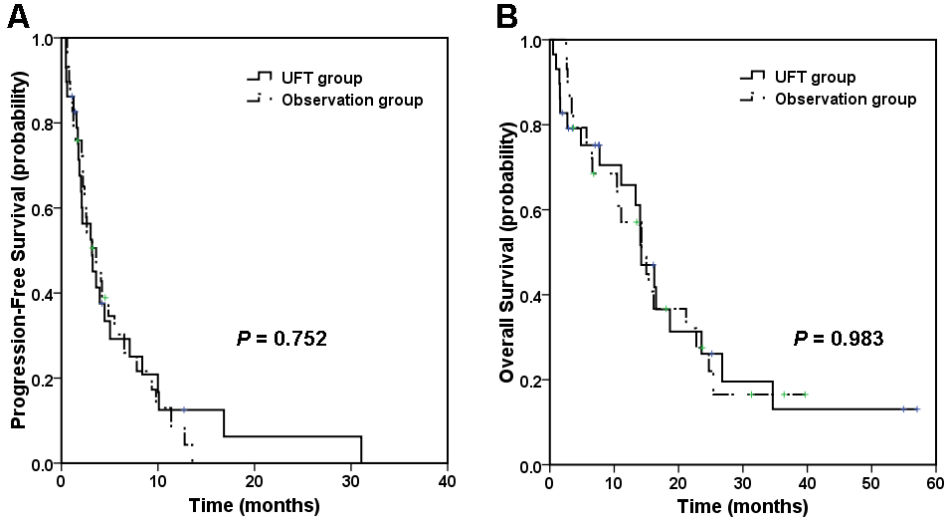
Kaplan-Meier estimates of progression-free survival (PFS) and overall survival (OS) **A**. Median PFS was 3.2 months in UFT group compared with 3.6 months in observation group. **B**. Median OS was 14.2 months in both UFT group and observation group.

After a median follow-up of 31.3 months (range, 1-57.1 months), 51 patients experienced disease progression, including 25 (89.3%) in the UFT group and 26 (89.7%) in the OBS group. The median PFS was 3.2 months (95% CI, 1.4-5.0) for the UFT group and 3.6 months (95% CI, 1.7-5.5) for the OBS group. No significant difference was observed between the two groups with the P value of 0.752 (Table 2, Figure 1A). The median OS was 14.2 months (95% CI, 11.1-17.3) with the UFT group and 14.2 months (95% CI, 8.2-20.3) with the OBS group (*P* = 0.983, Table 2, Figure 1B).

**Table 2:** Analysis of efficacy in full analysis set

	UFT group (*n* = 29)	OBS group (*n* = 29)	*P*
PFS			
Disease progression or death, No.	25	26	
Median (95% CI), months	3.2 (1.4 to 5.0)	3.6 (1.7 to 5.5)	0.752
OS			
Death, No.	19	21	
Median (95% CI), months	14.2 (11.1 to 17.3 )	14.2 (8.2 to 20.3)	0.983

Abbreviations: PFS, progression-free survival; OS, overall survival.

**Figure 2:**
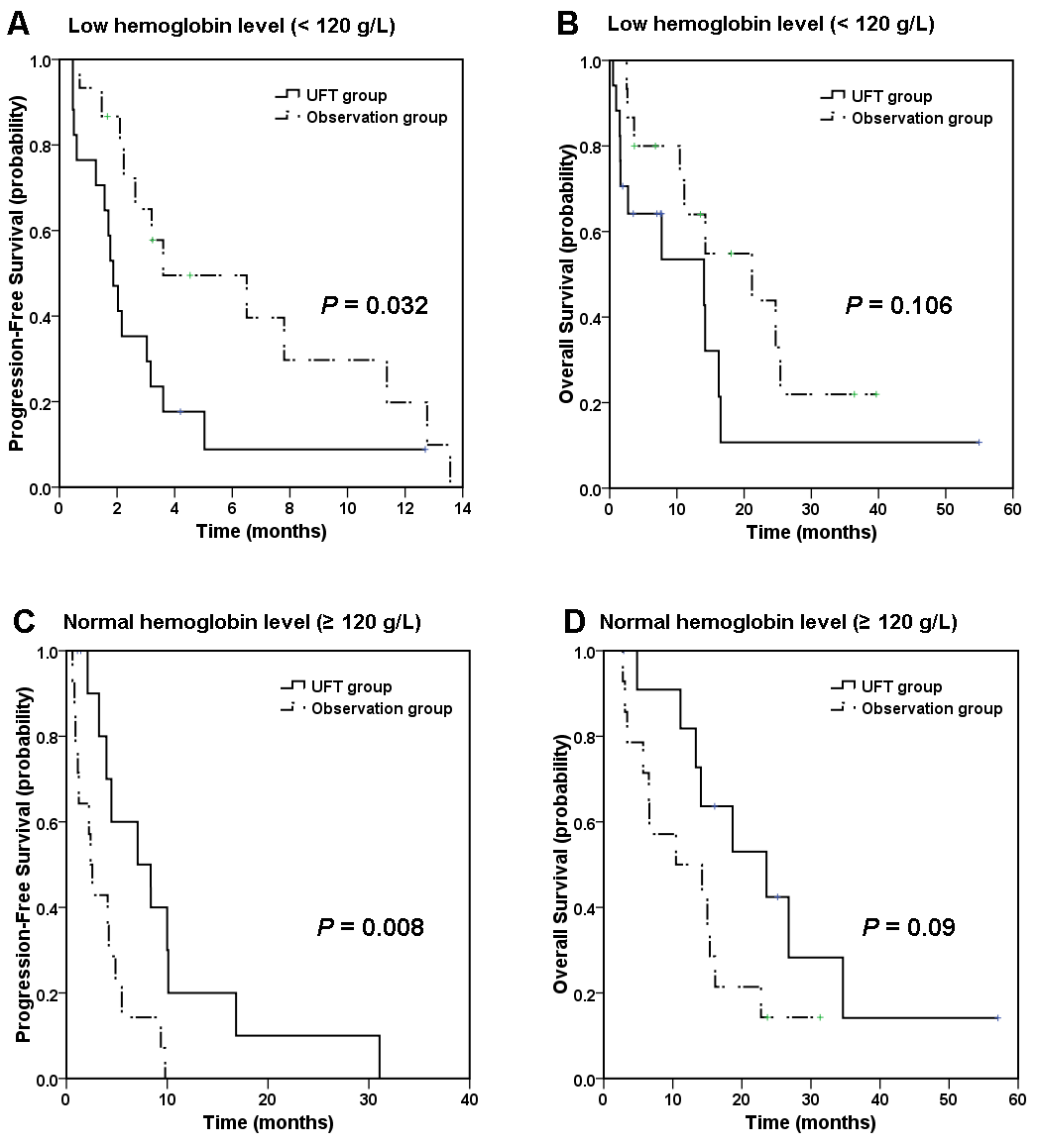
Kaplan-Meier curves of progression-free survival (PFS) and overall survival (OS) by baseline hemoglobin subgroup In patients with low hemoglobin level (< 120 g/L), **A**. median PFS was 1.9 months in UFT group compared with 3.6 months in observation group, and **B**. median OS was 14.0 months in UFT group compared with 21.2 months in observation group; In patients with normal hemoglobin level (≥ 120 g/L), **C**. median PFS was 7.1 months in UFT group compared with 2.4 months in observation group, and **D**. median OS was 23.6 months in UFT group compared with 10.5 months in observation group.

Baseline hemoglobin subgroup analysis showed that patients with low hemoglobin (< 120 g/L, *n* = 32) had a shorter PFS after the maintenance therapy (1.9 months in 17 patients of UFT group versus 3.6 months in 15 patients of OBS group, *P* = 0.032, Figure 2A), whereas patients with normal hemoglobin (≥ 120 g/L, *n* = 26) benefit from the UFT maintenance (7.1 months in 12 patients of UFT group versus 2.4 months in 14 patients of OBS group, *P* = 0.008, Figure 2C). Similar trend was also observed in the OS analysis. Patients with normal baseline hemoglobin had a better survival trend after the maintenance therapy (23.6 months versus 10.5 months, *P* = 0.09, Figure 2B), whereas patients with low hemoglobin did not (14. 0 months versus 21.2 months, *P* = 0.106, Figure 2D).

Stratified with the best efficacy in prior chemotherapy, CR/PR responders (*n* = 28) had no improvement in PFS and OS after the maintenance therapy compared with SD patients (*n* = 30), with a P value of 0.706 and 0.99.

### Safety

The most common adverse events with incidences of 10% or greater in the UFT group were leucopenia, anemia, thrombocytopenia, hyperbilirubinemia, fatigue, increased ALP, decreased appetite and abdominal distension (Table 3). Grade 3 to 4 toxicities in the UFT group were anemia, thrombocytopenia and diarrhea. Rare hematologic adverse events and gastrointestinal reaction (less than 10%) were reported in OBS group. Anemia and increased ALP in any grade occurred more often in the UFT group than in the OBS group (respectively, 41.4% versus 6.9%, *P* = 0.0049; 24.1% versus 0%, *P* = 0.01). Grade 3 diarrhea was increased in the UFT group, but this was not significant (6.9% versus 0%, *P* = 0.49). Only two patients required dose reduction of UFT because of severe diarrhea.

**Table 3:** Analysis of safety in full analysis Set

	No. (%)			
	UFT group (*n* = 29)		OBS group (*n* = 29)	
Adverse Event	Any grade	Grade 3 or 4	Any grade	Grade 3 or 4
Hematologic				
Leucopenia	7 (24.1)	0	2 (6.9)	0
Neutropenia	4 (13.8)	0	1 (3.4)	0
Anemia	12 (41.4)	1 (3.4)	2 (6.9)	0
thrombocytopenia	3 (10.3)	1 (3.4)	1 (3.4)	0
Non-hematologic				
Hyperbilirubinemia	3 (10.3)	0	0	0
Fatigue	3 (10.3)	0	0	0
Increased ALP	7 (24.1)	0	0	0
Elevated GGT	2 (6.9)	0	1 (3.4)	0
Decreased appetite	6 (20.7)	0	2 (6.9)	0
Diarrhea	2 (6.9)	2 (6.9)	0	0
Abdominal distension	4 (13.8)	0	1 (3.4)	0

Abbreviations: ALP, alkaline phosphatase; GGT, γ-glutamyltransferase

## DISCUSSION

To our knowledge, this is the first randomized phase II study evaluation of oral fluoropyrimidine maintenance after the first-line fluorouracil-based chemotherapy in metastatic gastric cancer patients. The majority of the patients received 5-day EOF regimen in our previous studies [14, 15] and obtained the PR or SD response. Following the consideration of all available information, a decision was taken to close the trial after the enrollment of 58 of the planned 120 patients had been achieved. We did not observe the improvement in PFS and OS with the continuation of UFT after the initial treatment though the safety profile was acceptable.

Although fluorouracil-based three-drug or double-drug regimens are widely accepted as the first-line treatment for advanced gastric cancer [2, 3], it remains to be determined whether the maintenance therapy should be administered or not. The concept of chemotherapy administration till the development of progressive disease is still widely accepted though there is no direct evidence obtained from randomized controlled study. But it is not tolerated for the advanced gastric cancer patients to receive more than 6-8 cycles of the combination therapy due to the cumulated toxicity or deteriorated performance status. Therefore, continuation of fluorouracil is an option both in clinical practice and in international phase III studies, such as the EXPAND study [16] and AVAGAST study [17]. Capecitabine and/or targeted agent were designed to be continued until disease progression after six-cycle chemotherapy of platinum plus fluorouracil in advance gastric cancer patients [16, 17]. However, our results challenged the fluorouracil maintenance treatment in non-selected patients. Similar finding was also reported in the phase III COIN study in colorectal cancer [18]. No improvement in OS was found for all patients with the continuous chemotherapy and survival benefit was only suggested in the subpopulation of patients with higher platelet [18]. These data, including the results of COIN study and ours, showed the importance of suitable patient identification in the application of maintenance treatment.

The striking outcome from the study is the significant correlation of a normal baseline hemoglobin level as a predictive biomarker for the maintenance therapy strategy. Nearly half of the patients in this study had normal hemoglobin level at baseline and these patients had substantially increased PFS (a 4.7-month increase with a P value of 0.032) when treated with UFT, and the survival curve also separated in the analysis of OS with a 13-month increase although the P value did not reach statistical difference. By contrast, the patients with low hemoglobin level (80~120 g/L) had inferior PFS and no improvement in survival. The hemoglobin level has previously been identified as a predictive and prognostic factor in advanced gastric cancer [19, 20]. And patients with low baseline hemoglobin level rarely benefit from second-line chemotherapy [20]. The mechanism could relate to several reasons, such as the treatment-induced myelosuppression, bleeding, nutritional deficiency and other cytokine-induced chronic disease, all contributing to poor performance status. If confirmed in further randomized phase III study, the easily measurable marker of the hemoglobin level would be a helpful and cost-effective predictive biomarker for identification of patients in whom maintenance therapy might be preferable in order to prolong the clinically favorable state.

As the first generation of the oral prodrug of 5-FU, UFT is commonly used in East Asia and many other countries except US [21, 22]. The second generation compounds exhibited stronger antitumor activity [23]. For example, capecitabine is activated preferentially in tumors because of the metabolized enzymes in converting into 5-FU in vivo [24]. And S1 contains 5-chloro-2,4-dihydroxypyridine (CDHP, a potent reversible inhibitor of 5-FU degradation) and potassium oxonate (OXO, 5-FU phosphorylation selective inhibitor which distributes much higher in gastrointestinal tract)to potentiate the antitumor activity and decrease the toxicity [25]. Both of these two drugs were proved to be effective in the initial treatment for advanced gastric cancer patients [26–28]. In further study design, both capecitabine and S1 might be the alternative option in the maintenance treatment after the initial 5-FU-based chemotherapy in metastatic gastric cancer.

Safety profile of UFT in this study exhibited that myelotoxicity was mild while the gastrointestinal reaction was the major toxicity, but no hand-foot syndrome was observed. This was consistent with the data in previous trials of UFT in patients with other solid tumors [7, 10, 29, 30]. It may because that the gastrointestinal adverse event, diarrhea for instance, was correlated significantly with the maximum plasma concentration and AUC_0-6h_ of 5-FU, while the hand-foot syndrome was characteristic to continuous intravenous infusion of 5-FU [29, 31]. The plasma concentration of 5-FU after oral administration of UFT had wide interpatient and intrapatient variations. The determination of plasma level used to be recommended [31], but it was not routinely taken due to the inconvenience. In our study, the plasma concentration was not accessed and limited data could be shown.

Although the maintenance therapy of UFT did not reach the primary objective in whole population in this trial, it is possible that benefit could be achieved by better selection of patient population and the new agent. The normal hemoglobin level at baseline is a potential biomarker separating patient subsets who do better from the UFT maintenance treatment. Further randomized phase III study may help identify the role of oral fluoropyrimidine by considering the use of capecitabine or S1 in proper patients with normal hemoglobin.

## MATERIALS AND METHODS

### Patients

Patients aged 18-75 years with histologically or cytologically confirmed metastatic gastric adenocarcinoma received first-line chemotherapy with fluorouracil-based regimen (ECF/EOF or FOLFOX/XELOX regimen, 3-week regimen for 6 cycles and 2-week regimen for 12 cycles). Patients with stable disease or a better response with an Eastern Cooperative Oncology Group performance status (ECOG PS) of 0 to 2 and life expectancy of ≥ 3 months were eligible. Patients were required to have adequate bone marrow (neutrophil ≥ 1.5 ×10^9^/L, hemoglobin ≥ 80 g/L, platelet ≥ 75 ×10^9^/L), hepatic, and renal function. Patients with brain or meninges metastases, bowel obstruction, symptomatic peripheral neuropathy, severe cardiac disease or uncontrolled infection were excluded. The study was approved by the independent ethics committee of Fudan University Shanghai Cancer Center, Shanghai, China (Approval number IRB 090875-12) and registered at ClinicalTrials.gov (NCT02903498). The study was carried out in accordance with the Declaration of Helsinki. All patients provided written informed consent before study entry.

### Treatment

All enrolled patients were randomly assigned at a 1:1 ratio to receive either oral UFT or just observation (OBS), stratified by the best efficacy of the prior chemotherapy (CR+PR/SD). Patients received oral UFT (360mg/m2 × 2 weeks) every 3 weeks until disease progression or unacceptable toxicity, or consent withdrawal. The daily doses of UFT were divided into three doses administered 8 hours apart and taken together along with water. Patients were instructed to avoid food consumption between 1 hour before and 1 hour after each dose.

The primary endpoint was progression-free survival (PFS); the secondary endpoints were overall survival (OS) and safety. PFS was defined as the duration from the time of random assignment to the time of disease progression or death whichever occurred first or censored at the last follow-up visit.

### Dose modification

Dose adjustments were made based on the worst grade of toxicity encountered during the previous cycle. For hematological toxicities, the dose of chemotherapeutic drugs was reduced in the following cases: grade 4 neutropenia or leukopenia; grade 3 or greater febrile neutropenia; grade 3 or greater thrombocytopenia. For nonhematological toxicity, the dose was reduced when grade 3 or greater toxicities occurred (except for alopecia). The dose was reduced by 25% of the starting dose. If a patient required more than three successive dose reductions, therapy was discontinued.

Treatment was delayed until the absolute neutrophil count was ≥ 1.5×10^9^/L and platelet count was≥ 75×10^9^/L, and recovery to grade ≤ 1 for nonhematological toxicities (with exception of alopecia). The maximum authorized delay is of 2 weeks.

### Assessments

Pretreatment assessment included a detailed medical history, physical examination, routine laboratory tests, and performance status. Laboratory evaluation included a routine blood count, urinalysis, and electrolyte, renal, and liver function tests.

Radiographic scans (computed tomography or magnetic resonance imaging) for efficacy evaluation were conducted at baseline and every 6 weeks thereafter according to the RECIST 1.0 guidelines. The best overall response was reported. Survival status was assessed every 3 months after discontinuation of study treatment.

Adverse events and concomitant medications were recorded at the end of each cycle. Toxicity was evaluated and graded according to the National Cancer Institute Common Terminology Criteria for Adverse Events, version 3.0.

### Statistical analysis

This phase II study was designed to assess the improvement of PFS with maintenance UFT. We supposed that the UFT maintenance could improve the median PFS from 1.25 months to 3 months, compared with the observation group. Then, 120 patients (60 patients in each group) were needed with an exact significance level of *p* = 0.05 and a power of 90%, considering 10% drop-off.

Quantitative variables were compared between groups using two-sample *t* test or Wilcoxon rank sum test. Pearson Chi-square test or Fisher's exact test was used to analyze categorical variables, whenever appropriate. Survival function of time-to-event end points was estimated by using the Kaplan-Meier method. The log-rank test was used for comparisons of PFS and OS between the two groups. All statistical analyses were two-sided. The statistical significance level was set at .05. The CI was set at 95%. All statistical analyses were performed using SPSS software (version 18.0).

## Abbreviations

UFT: Uracil and tegafur
PFS: progression-free survival
OS: overall survival
ECF: epirubicin, cisplatin and continuous infusion of 5-fluorouracil
EOF: epirubicin, oxaliplatin and continuous infusion of 5-fluorouracil
EOX: epirubicin, oxaliplatin and capecitabine
FOLFOX: oxaliplatin, leucovorin and continuous infusion of 5-fluorouracil
XELOX: capecitabine and oxaliplatin
TKIs: tyrosine kinase inhibitors
5-FU: 5-fluorouracil
ECOG PS: Eastern Cooperative Oncology Group Performance Status
OBS: observation
RECIST: Response Evaluation Criteria in Solid Tumors
CR: complete response
PR: partial response
SD: stable disease
ALP: alkaline phosphatase
CDHP: 5-chloro-2,4-dihydroxypyridine
OXO: potassium oxonate.
